# Homing wellness: can narrative design transform living spaces for people with dementia into engaging environments enabling communication?

**DOI:** 10.3389/fpubh.2023.1198253

**Published:** 2024-01-15

**Authors:** Loukia Minetou, Avraam Chatzopoulos, Anastasios Tzerachoglou, Georgios Priniotakis, Joost van Hoof, Emmanouela Sfyroera, Zoe Georgiadou, Styliani Tyrovola, Christos Drosos

**Affiliations:** ^1^Centre for Environment Dementia and Ageing Research, Faculty of Social Sciences, University of Stirling, Stirling, United Kingdom; ^2^Department of Industrial Design and Production Engineering, School of Engineering, University of West Attica, Athens, Greece; ^3^Research Group of Urban Ageing, Faculty of Social Work and Education, The Hague University of Applied Sciences, The Hague, Netherlands; ^4^Faculty of Spatial Management and Landscape Architecture, Institute of Spatial Management, Wrocław University of Environmental and Life Sciences, Wrocław, Poland; ^5^Department of Interior Architecture, School of Applied Arts and Design, University of West Attica, Athens, Greece

**Keywords:** dementia, narrative design, spatial objects, care home design, communication in dementia care, embedded technology, interactive textiles, multi-sensory experience

## Abstract

Interactive design is an emerging trend in dementia care environments. This article describes a research project aiming at the design and development of novel spatial objects with narrative attributes that incorporate embedded technology and textiles to support the wellbeing of people living with dementia. In collaboration with people with dementia, this interdisciplinary research project focuses on the question of how innovative spatial objects can be incorporated into dementia long-term care settings, transforming the space into a comforting and playful narrative environment that can enhance self-esteem while also facilitating communication between people living with dementia, family, and staff members. The research methodologies applied are qualitative, including Action Research. Participatory design methods with the experts by experience—the people with dementia—and health professionals have been used to inform the study. Early findings from this research are presented as design solutions comprising a series of spatial object prototypes with embedded technology and textiles. The prototypes were evaluated primarily by researchers, health professionals, academics, and design practitioners in terms of functionality, aesthetics, and their potential to stimulate engagement. The research is ongoing, and the aim is to evaluate the prototypes by using ethnographic and sensory ethnography methods and, consequently, further develop them through co-design workshops with people living with dementia.

## 1 Introduction

According to the WHO (1), ~55 million people live with dementia worldwide, and this number is forecast to increase in the years to come. In North-Western Europe, a significant percentage of people with dementia reside in institutional care settings, including care and nursing homes. In the UK alone, 40% of people with dementia over 65 live in care homes (2, 3). After a prolonged period of institutionalization, many older people with dementia feel lonely and socially isolated and have symptoms of depression (4), which is related to a plethora of social, physical, and psychological problems.

In recent years, research on the impact of design that aids independence and enhances self-esteem and quality of life has increasingly demonstrated the supportive role of the care home environment towards a good life (5, 6). Recent research begins to show the importance of the local environment in social health (7). The built environment is one of the factors determining the quality of life of people with dementia, who may face difficulties in recognizing and navigating the environment of the care home or accessing opportunities for social interaction and participation in daily activities (8–12). Design features, for example, the use of light, color, and contrast that compensate for sensory changes in people with dementia and aid them in way-finding, are examples of developments in care home design accepted as elements of best practice (13, 14). Non-clinical practices that have been studied in relation to the wellbeing and social engagement of people with dementia include multi-sensory stimulation environments (MSEs) (15–17). However, MSEs limited to a specific room in care homes might not effectively meet the needs of people with dementia (18). Research indicates that a stronger focus on sensory-built environment design elements is needed, especially for addressing the needs of people in the later stages of dementia (11, 12).

Furthermore, design innovations include attempts to embed technology in products used by people with dementia as well as in their living environment. Such projects range from leisure products to smart home technologies and wearables intended to support daily needs and tasks (19–24). Lately, the emergence of a broad range of assistive technology products for people with dementia, positively received by users, has been observed despite the lack of hard quantitative evidence. For example, HUG, a huggable soft object with embedded technology (25), and PARO, a therapeutic robot seal (26), were introduced as non-pharmacological treatments.

Even though studies investigating the impact of handling sensory objects on the wellbeing of people with dementia suggest their positive value in providing opportunities for engagement, storytelling, and social interactions through verbal and non-verbal communication (25, 27–30), there is a lack of research on large sensory spatial products, especially with embedded technology. The research described in this study, titled Homing Wellness, examines if embedded technology can be used in spatial objects to enhance the sensory experience of people with dementia and if such objects can interrelate to create a narrative spatial network potentially supporting communication. The emphasis of this interdisciplinary investigation is on examining art-led *narrative design* in the creation of sensory spatial objects that can aid residents, staff, and family members in expressing past stories or inventing new ones (31).

The term narrative design is informed by scenographic principles established in theater, which utilize design to tell stories and support audiences in engaging in narratives by employing all the senses. Narrative design incorporates elements (for instance, objects, lighting, textures, sounds, and smells) intended to encourage storytelling by those using, moving through, or observing the space. Taken from the concept of narrative design in theater—also known as scenography—it refers to the configuration of space and its contents to reinforce the narrative of the story being told (32).

The theoretical framework of this study is based on the idea that people interact with the world through all of their senses. Communication is usually studied mostly on the basis of optical (visual) and acoustic (audio) channels, although a person can interact through the full range of the five senses, which are co-organized (interrelated), leading to an effect of “intersensoriality” (33). This organization of the senses can be seen as an interactive web that allows the senses to work together. This approach allows us to see the experience of people with dementia in a holistic way, emphasizing the significance of the haptic and olfactory channels of communication as a means of enhancing interaction. Connected and interacting in this way, people with dementia can feel better supported, especially in the late stages, as they are often immobile and, in some cases, have no verbal communication.

The study's approach aligns with concepts of embodiment and embodied personhood claiming that when dementia has progressed, selfhood is still alive and demonstrates itself through the body (34–36). These concepts are based on the idea that human beings create systems of navigation—body schemas—that allow them to interact with their surroundings (37). These systems come into play even when cognition is not actively participating in our decision-making, thus facilitating ways to relate to our environment. Adhering to these theories, we can understand how people with dementia can relate to their surroundings through the mind and body as a whole. The study also adopts concepts deriving from Kitwood's notion of relational hypostasis of personhood. For Kitwood, personhood is a status one attains because of the way one is seen by others (38, 39). Communication in dementia long term care settings (LTC) is vital, for personhood needs the *other* to be sustained. Thus, by offering triggers for interaction, opportunities emerge for meaningful social engagement and for maintaining the status of a unique human being who is worthy of respect and dignity. The project also incorporates ideas on the importance of narratives to express personal identity and acquire agency for people with dementia (40). There is evidence that sensory engagement, especially through storytelling, benefits communication and improves quality of life for people with dementia. Research on the benefits of a free-form storytelling intervention for persons with dementia residing in care homes has shown this to include enhanced pleasure, more frequent social interactions and communication, and more positive staff views of residents with dementia (41, 42). Such findings suggest that giving people with dementia the opportunity to continually access objects that prompt storytelling is important for the improvement of communication and quality of life. This study aims to create designs for spatial objects that will provide people experiencing dementia with possibilities for individual or collaborative storytelling.

## 2 Research aims, objectives, and questions

In collaboration with people with dementia and the health professionals of two partner LTC settings, Homing Wellness focuses on the question of how interactive spatial objects can be incorporated into dementia care homes, transforming the space into a comforting and playful environment that can enhance self-esteem, facilitate communication, and improve quality of life for both residents and staff members. The study aims to develop a series of spatial objects with embedded technology, examine the narrative possibilities of individual spatial sensory objects, and also examine their narrative potential as an interrelated network. The spatial objects combine functionality and aesthetics while incorporating embedded technology and soft textures implemented by textiles. A flexible set-up of the embedded technology allows for personalization. The objects, aiming to enhance a sense of empowerment and agency by providing opportunities for verbal and non-verbal storytelling (35, 40), are designed to be accessible and easy to use by people with dementia.

This research aims to apply the following actions.

1. To create designs for a range of spatial objects as part of the research questions (a) how spatial objects with embedded technology can facilitate self-expression and stimulate communication to enhance social connectivity and (b) how to provide sources of interest and occupation to enhance a sense of pleasure and wellbeing.
2. To develop and produce a series of spatial object prototypes.
3. To install the prototypes in the partner LTC settings, collect data from their use by people living with dementia, and utilize the data to improve the design in consequent research cycles.

The involvement of experts by experience, carers, and health professionals has shaped the underpinning research, attempting to ensure that the design derives from and responds to the needs and experiences of people with dementia. This article reports on the development and creation of the designs and the production of the spatial objects' prototypes (actions 1 and 2). The installation and data collection (action 3) are ongoing, and a report will follow after the completion of the above actions.

## 3 Methodology

**Figure 1:**
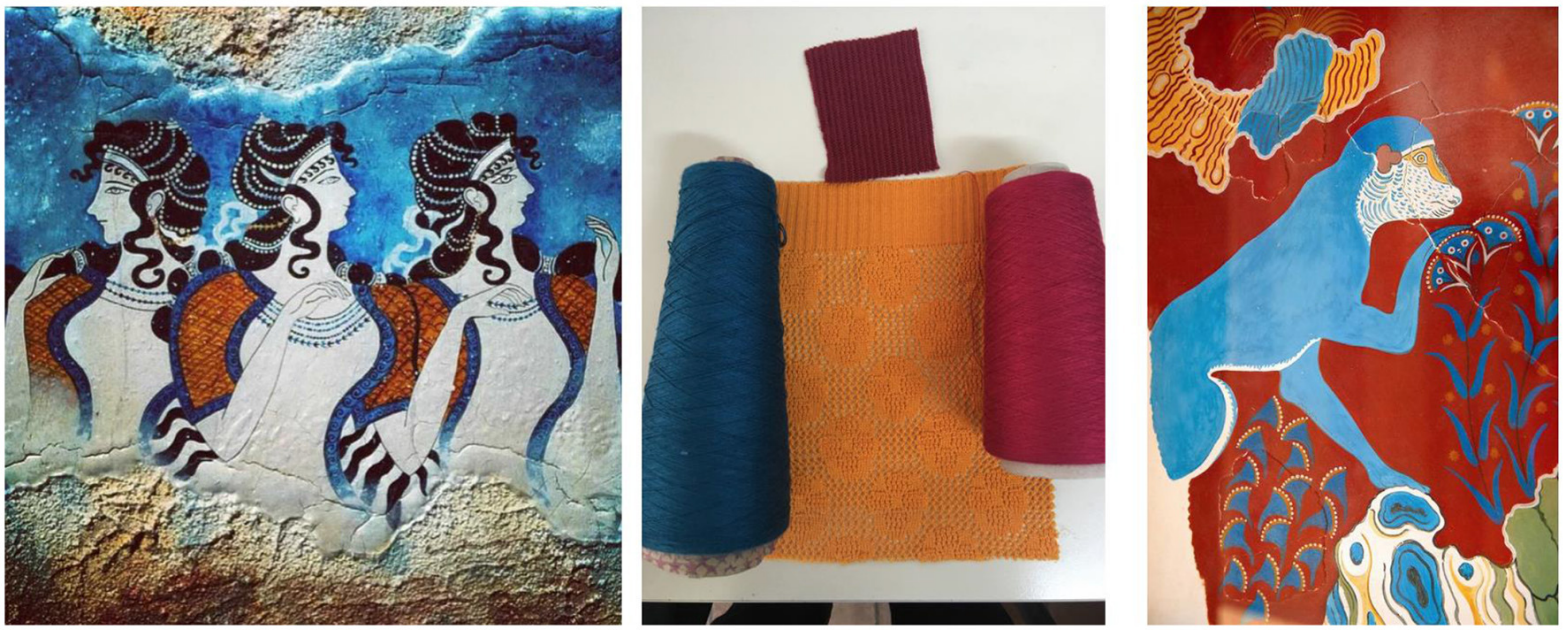
Minoan palace wall paintings used as inspiration.

The creative process uses metaphors from mythology, which could elicit mnemonic references. Myths are semiotic vehicles and help people make sense of the world in which they live (43, 44). In this particular case, the Myth of Ariadne's Clew was used as a reference point for the research team to identify the context for the design approach (Figure 1). The myth is used to guide the design research process and the implementation of the design product, as well as a metaphoric clue to support finding storytelling-based communicative patterns.

Adopting a participatory methodology to involve stakeholders throughout the research process, the study is guided by health professionals and includes co-design with persons with dementia in developing new narrative design ideas for care homes (45, 46).

**Figure 2:**
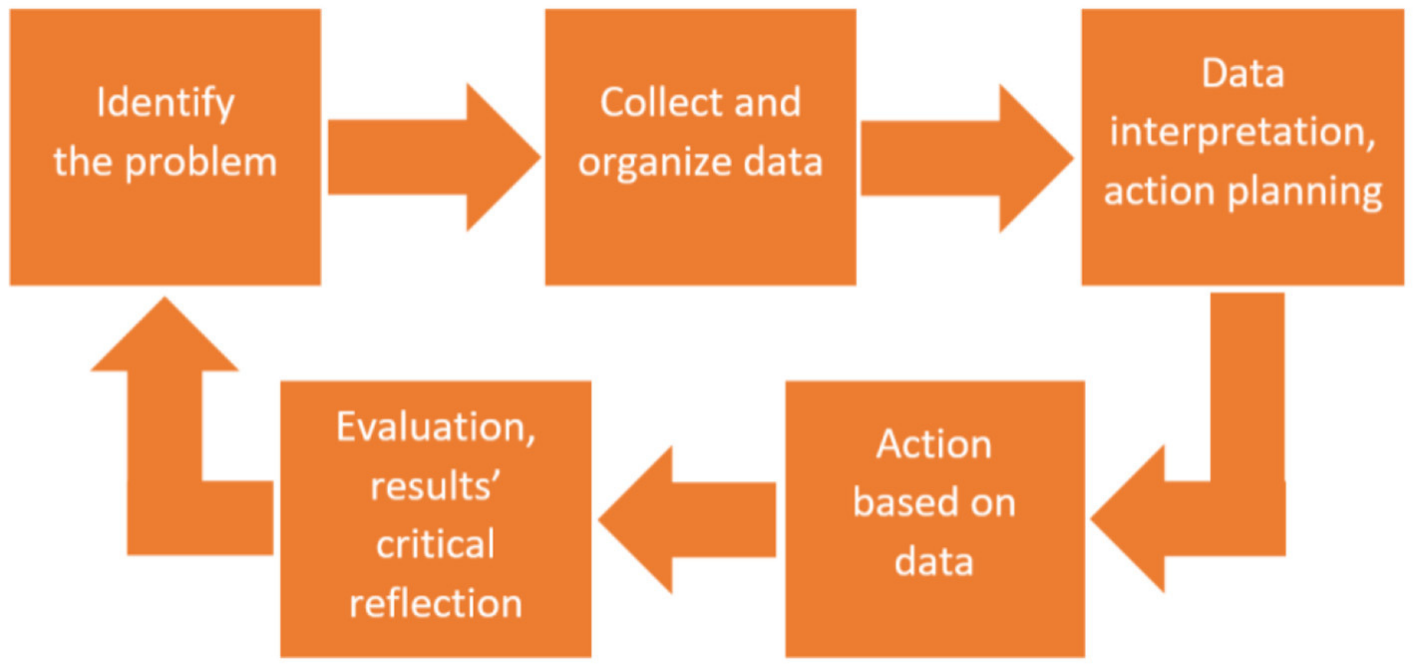
Action research five phases' model.

The research utilizes action research (AR) as the key tool for fulfilling the research aims and objectives. AR allows researchers and participants to collaboratively link theory to practice to contribute effectively to actions that drive social change (47, 48). AR employs data to solve practical problems in a particular arena in order to improve it while allowing researchers to understand their practices and generate knowledge (49, 50). AR models may vary according to context, place, and space (51, 52) with most of them accepting the spiral (or circular) process. In this research, the five-phase AR model suggested by Greeff and Coetzee (51) is employed, involving: (phase 1) identification of the problem; (phase 2) data collection and organization; (phase 3) data interpretation and action planning; (phase 4) action implementation based on data findings; and (phase 5) evaluation of the results and critical reflection (Figure 2).

**Figure 3:**
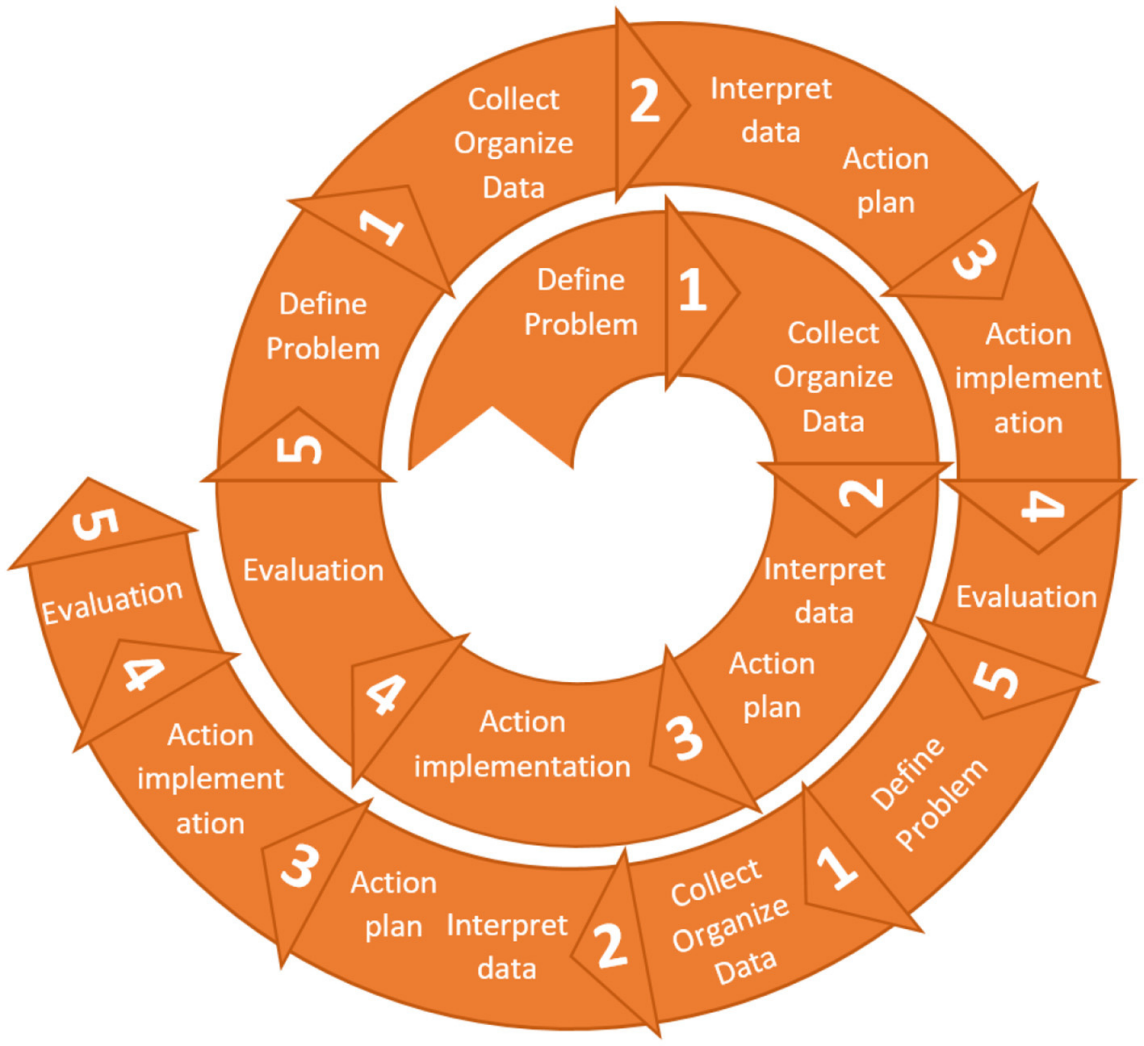
Proposed action research cycles and phases.

All of the above five phases mark a cycle of AR, with the knowledge gained from it feeding the next AR cycle where the research process is repeated, hence the AR's cyclical or spiral process. A three-cycle, five-phase (per cycle) model, adapted to the purposes of this research, is applied (Figure 3).

### 3.1 Action research cycle 1

Phase 1–2: A scoping literature review was conducted to identify previous research and practice in spatial multi-sensory interventions and their implications for the quality of life of people with dementia. A specific focus was placed on social interaction through opportunities for narration and storytelling. The review investigated existing research and the impact of multi-sensory interventions, assistive technology, and gaps in environmental design research supporting care for people with dementia.

Phase 3–4: The results of the literature review were analyzed and incorporated into the following steps: (1) the researchers' brainstorming to generate initial ideas for the design of sensory textiles and spatial objects; (2) the creation of the guidelines for (a) the semi-structured interviews with the health professionals and (b) the co-design workshops with people with dementia; and (3) the drafting of the design guidelines for the prototypes. These guidelines are based on the Fleming–Bennet principles and the values suggested in the Dignity Manifesto of Design (53, 54).

Phase 5: An initial consultation with the health professionals provided expertise to evaluate and further shape the design guidelines: (1) to be accessible people in different stages of dementia; (2) to feel comfortable (safe, well-proportioned, and ergonomic design); (3) to support feelings of confidence/familiarity and homeliness with significant meaning (familiar details, imagination triggers, and storytelling); (4) to provide a multi sensory experience (use of olfactory, tactile, and audio-visual elements); (5) to stimulate and relax (with the animated elements, music, and storytelling); (6) to foster a sense of control (easy to use interface and accessible technology); (7) to provide a trigger for interaction (embedded technology, proximity sensors, and attractive design); (8) to support feelings of safety and security (compatible with age and with proper usage specifications); (9) to make use of strengths (motion responsive interface); (10) to support the continuing use of the senses (different materials/textures, variety of shapes, animated elements, and possibility for exploration), and (11) to provide opportunities for communication/stimulate social contact (through storytelling triggers). These areas were covered by the overlapping applications of technology (communication, relaxation, multi-sensual, interactivity, sense of control, continuing use of senses, and mobilization), textures/colors/shapes (exploration, communication, multi-sensual, and continuing use of senses), and design aesthetics (familiarity and home-likeness).

### 3.2 Action research cycle 2

Phase 1–2: A series of semi-structured interviews were conducted with the health professionals of the two partner LTC settings, discussing the needs of residents in their LTC settings, their experiences and knowledge of sensory interventions, storytelling techniques, and ways of enabling communication with people with dementia. Present gaps in design features that could facilitate engagement and communication were also discussed. These discussions have shaped the initial ideas for the design, which in turn were visualized and communicated several times during the action research circles with the healthcare professionals.

Phase 3–4: Based on the previous phase's findings, co-design workshops with researchers, designers, and engineers involved in the project took place to draft initial ideas in line with the research's objectives. Guided by a participatory design approach, the original planning included a series of co-creation workshops with people with dementia recruited from the partner care homes while progressing through the different stages of the design.

**Figure 4:**
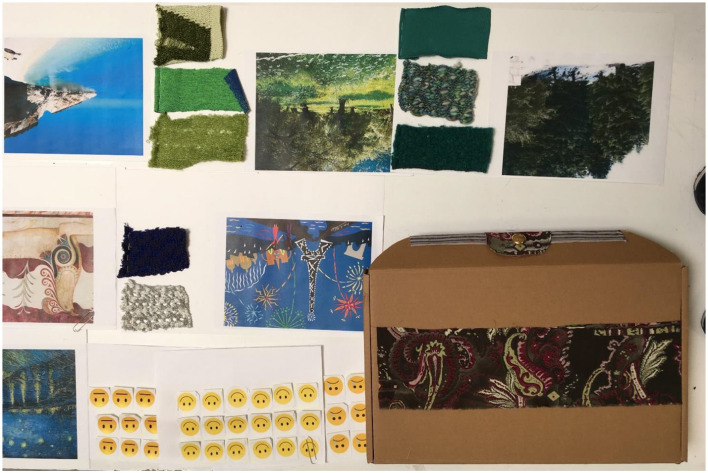
Box with sensory samples.

However, due to COVID-19 restrictions, the planned face-to-face workshops did not take place, and remote consultations were planned. Discussion sessions were held with health professionals and design experts experienced in working with vulnerable groups, aiming to maximize best practice knowledge for co-designing with people with dementia. The results of these sessions informed the production of a comprehensive project presentation and a set of two boxes with sensory samples (visual, tactile, and acoustic) to facilitate the remote involvement of people with dementia in the design of the prototypes (Figure 4).

Phase 5: Evaluation of the project presentation and the boxes with sensory samples by the health professionals. The boxes were sent to individuals to get their opinions on the design's initial ideas.

### 3.3 Action research cycle 3

Phase 1–2: Based on the last phase's findings, a series of three prototype spatial objects with embedded technology and textiles were designed and developed to support the wellbeing of people with dementia. A follow-up consultation with the health professionals of the care homes provided expertise in identifying problems and areas to amend the design.

Phase 3–4: The prototypes were completed, and preliminary tests were carried out by the research team and university staff. During this phase, discussions with the care home experts took place in order to form an overall spatial design involving the arrangement of the objects within the space, aiming to maximize the environment's narrative potential. To achieve the utilization of the spatial objects as part of such a narrative network, it was necessary to study the space and envision possibilities for the installation of the spatial objects. Homing Wellness aims to create design elements that are absorbed by the built environment, are permanent, and that people can access at any time. To meet this design objective, further suggestions for the spatial objects' installation in the care homes will be provided by people with dementia and the LTC settings' managers.

Phase 5: The project is ongoing, and the evaluation of the spatial objects is planned to take place after their installation in the partner LTC settings. This stage will be informed by sensory ethnography (SE). SE is suitable for research that attempts to understand the experiences of people with dementia, as it focuses on recording in-the-moment experiences from the perspective of the users by utilizing “sensory ways of knowing” through the sensations of touch, hearing, and smell (55, 56). Guided by SE, alongside traditional ethnographic methods for data collection such as semi-structured interviews, video interviews, and participant observation notes, this stage will involve the following dementia-inclusive methods: (a) interviews with the support of common objects and clothing to facilitate conversations (57–59); (b) walking interviews suggested in dementia research to facilitate dialogues inspired by their surroundings (60); (c) photovoice allowing the participants to produce their own photographs and filmography to capture sources of interest (61); and (d) video-elicitation interviews involving previously recorded video clips to prompt the participants to more extensively discuss certain issues (62).

### 3.4 Research ethics

Following the rules of the Helsinki Declaration, the approval (32538–24/03/2023) by the Research Ethics Committee of the University of West Attica in Athens has been received. The data collected from people living with dementia, carers, and relatives are handled and stored according to the Data Protection Law.

### 3.5 Research participants

The research is carried out with the participation of health professionals, a group of advisors with lived experience, and design practitioners. The overall evaluation will be carried out during the forthcoming next stage of the research with people with dementia, their relatives, and their carers. Fifty people participated in the research program, 30 of whom were people with dementia, and 10 were nurses/carers of people with dementia. The recruitment was conducted through the partner LTC settings, which are located in Greece (one in Athens and one on the island of Kefalonia).

### 3.6 Data collection

In the context of AR, mixed qualitative methods have been used to collect data for the initial design and production of the prototypes. Data were collected during the three first cycles of the AR, provided by the health professionals of the partner LTC settings in video-recorded semi-structured interviews as well as through discussions with design practitioners and advisors with lived experience. The data acquired so far have been utilized in the ideation and design of the spatial objects during the AR cycles. The biomedical model focusing on quantitative data collection, which has been used until recently in dementia research, restricts studies to a clinical approach and leaves less room for flexibility than is necessary. It is increasingly recognized that qualitative research foregrounds the voices of people living with dementia. There is a need for a co-produced and flexible approach to research design and data collection in interdisciplinary dementia studies (63, 64).

As discussed in section 3.3, for the next stage of the research, qualitative data will be collected from people living with dementia and their carers using semi-structured interviews and other dementia-inclusive methods such as photo-voice, walking interviews, and video-elicitation interviews. Such qualitative methods are dementia-inclusive and therefore suitable to understand the experiences of people living with dementia themselves without prioritizing the opinion of proxies. However, a wider implementation of the project is planned, which will enable the collection of quantitative data.

## 4 Results

Outcomes regarding the spatial object prototypes are presented here as design solutions involving embedded technology. The three spatial object prototypes developed are an interactive armchair, a wall picture frame, and a portable cover for a handrail.

**Figure 5:**
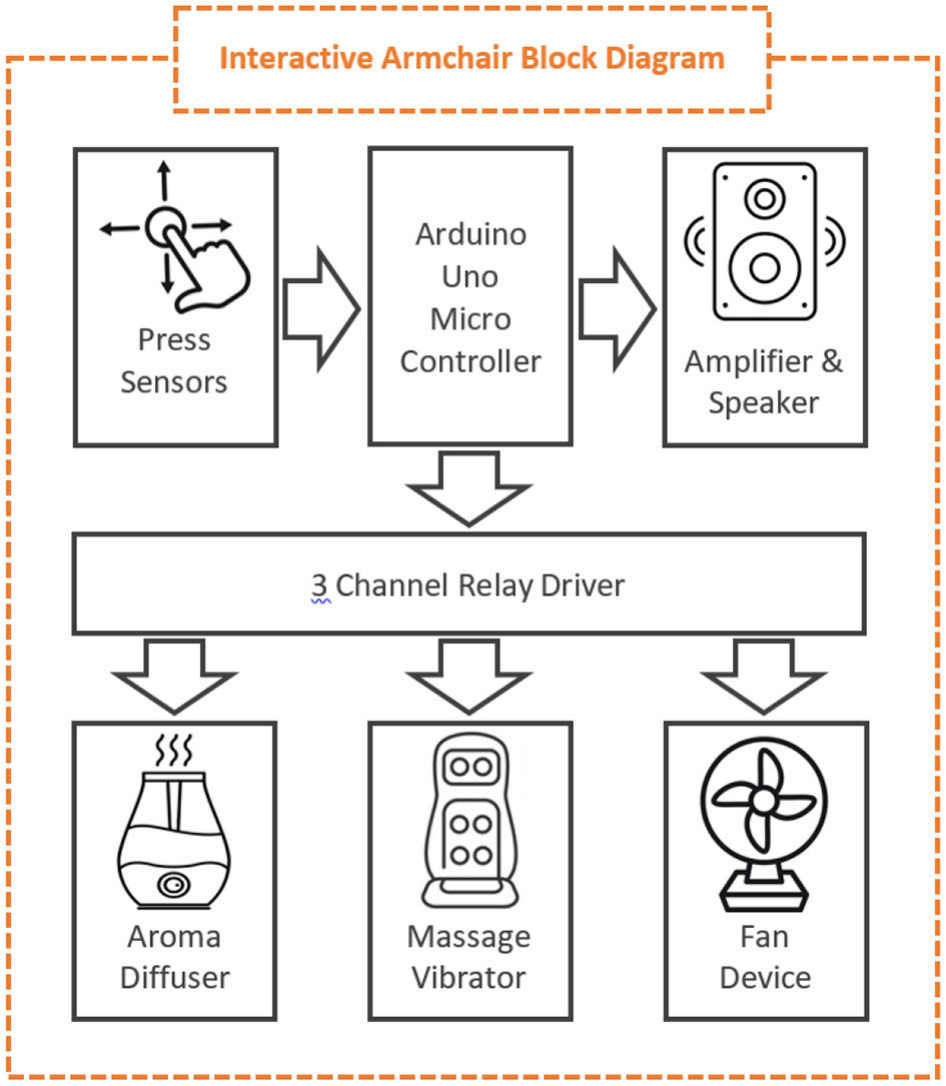
Interactive armchair block diagram.

**Figure 6:**
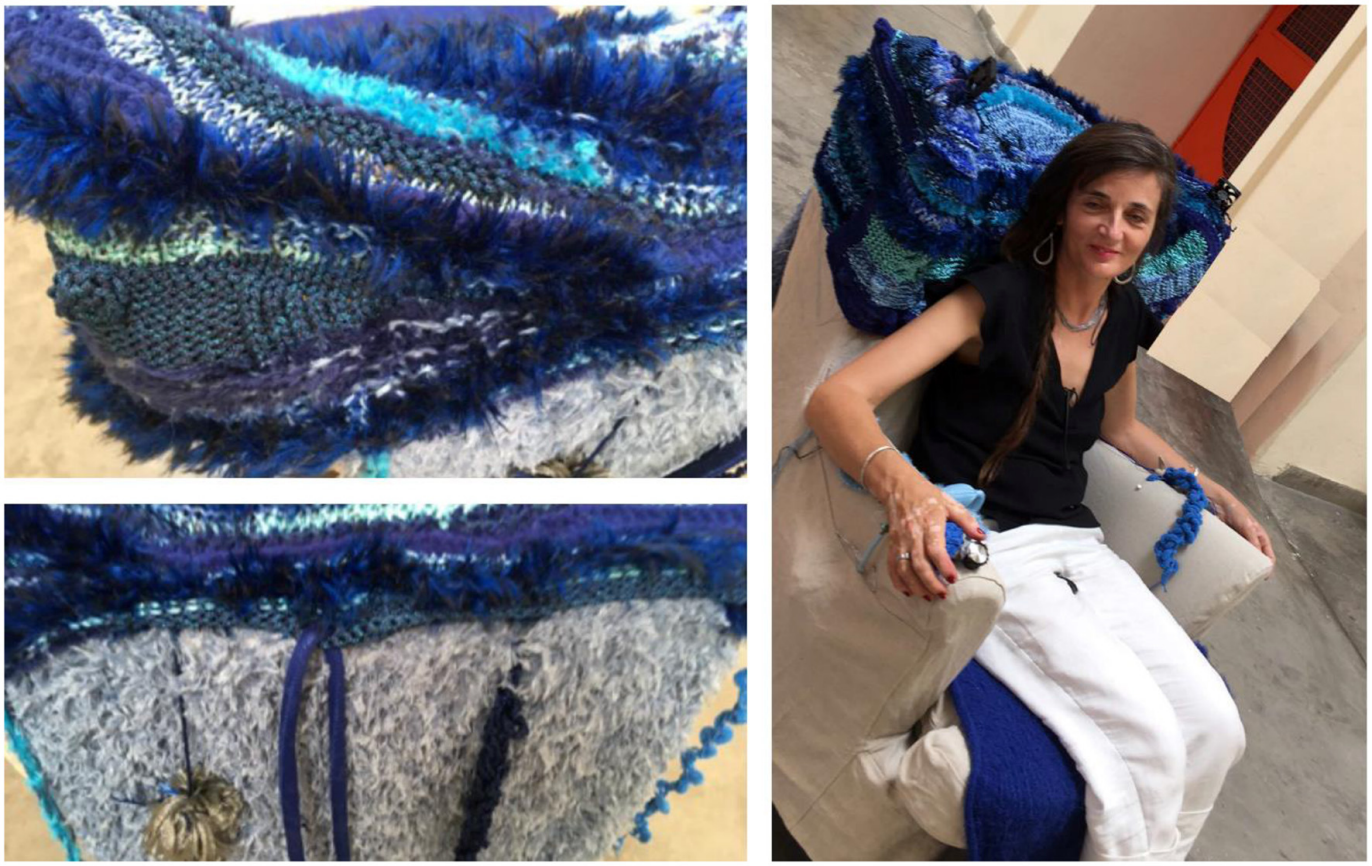
The sea-themed interactive armchair prototype (the person pictured is one of the authors), work in progress.

An *interactive armchair* (Figures 5, 6). This is an ordinary armchair equipped with multiple sensors (buttons, switches, and trimmers) and actuators (fan, aroma diffuser, massage vibrator, and speakers) covered with sea-themed fabrics.

The operating switches have an intuitive interface design and are located on the arms of the chair for easy access. The design of the armchair aims to offer the experience of nature by simulating the sounds, freshness, and breeze characteristic of the seaside. The sea theme was initially suggested by the advisors with lived experience. There were suggestions from health professionals that the experience of using the armchair could provide engagement for people whose mobility is affected and who cannot go for outdoor walks.

**Figure 7:**
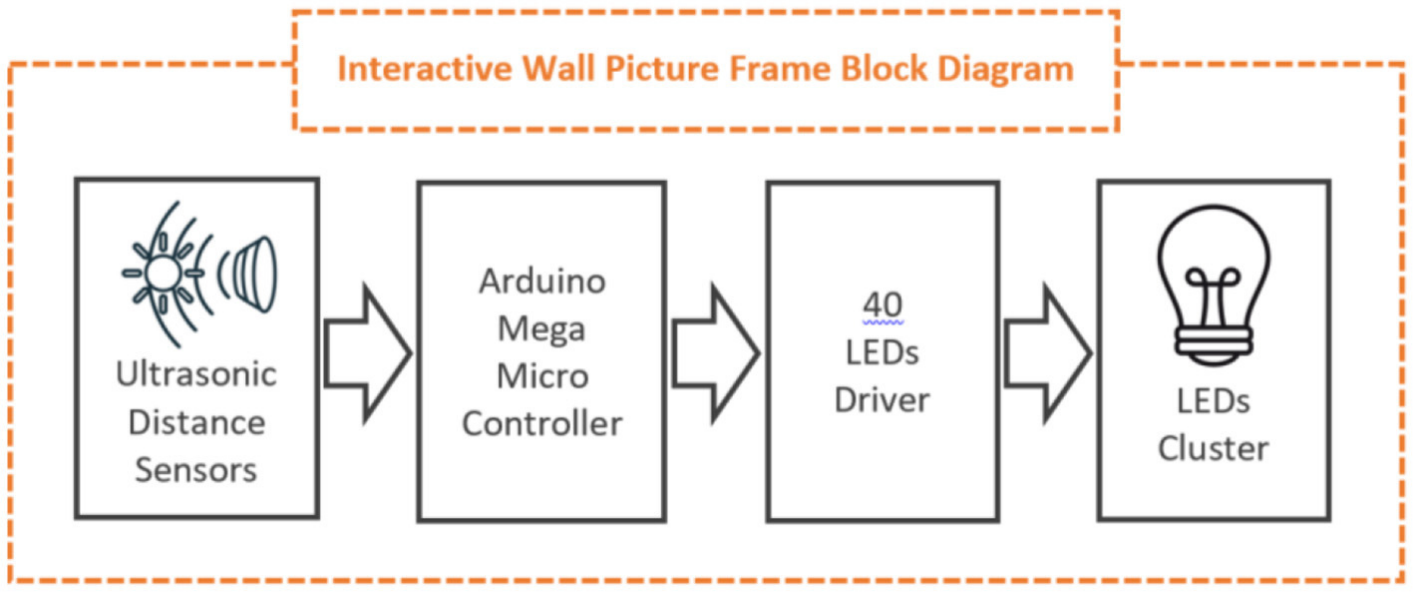
Interactive wall picture block diagram.

**Figure 8:**
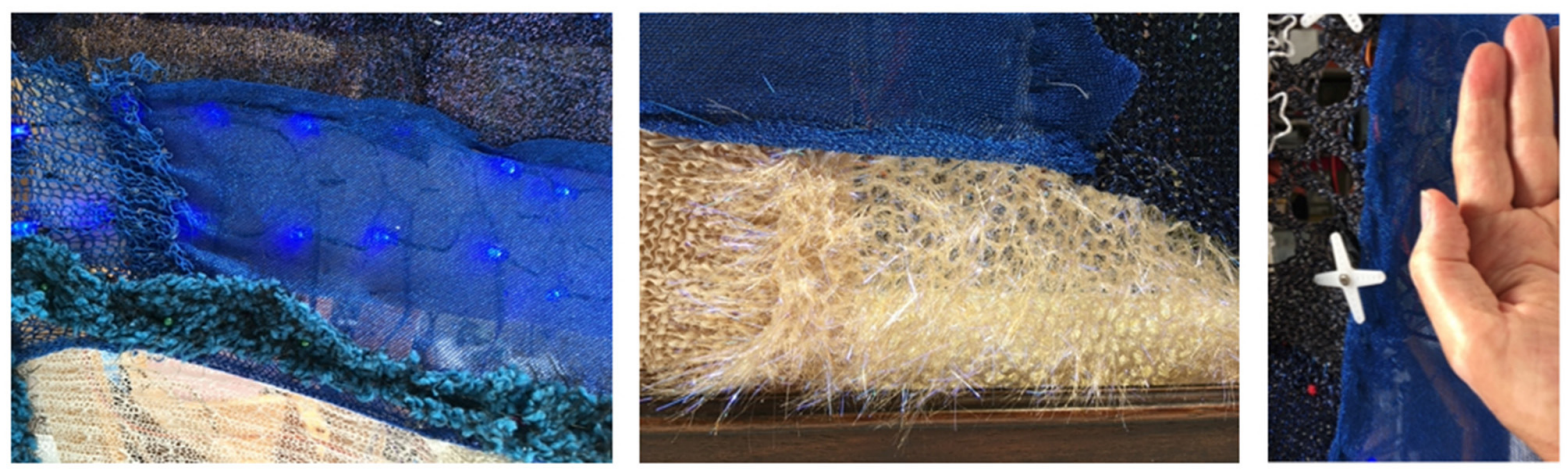
Interactive wall picture prototype at an early stage of development.

**Figure 9:**
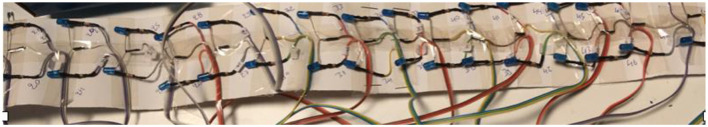
Interactive wall picture prototype at an early stage of development.

An *interactive wall picture frame* (Figures 7–9). This wall picture frame can alternate visual patterns, activate motion on different parts of the picture, and produce specially composed sounds. It is equipped with three ultrasonic proximity sensors, each of which performs a different scenario depending on how close someone is. For example, the first sensor varies the blue color brightness of an embroidered area of the frame (Figures 8, 9). When someone is further away, the brightness is at its maximum. As the person approaches, the brightness decreases. The second sensor produces sounds and music as they approach. The third sensor generates motion in a specific feature (such as a flower) embedded in the frame.

**Figure 10:**
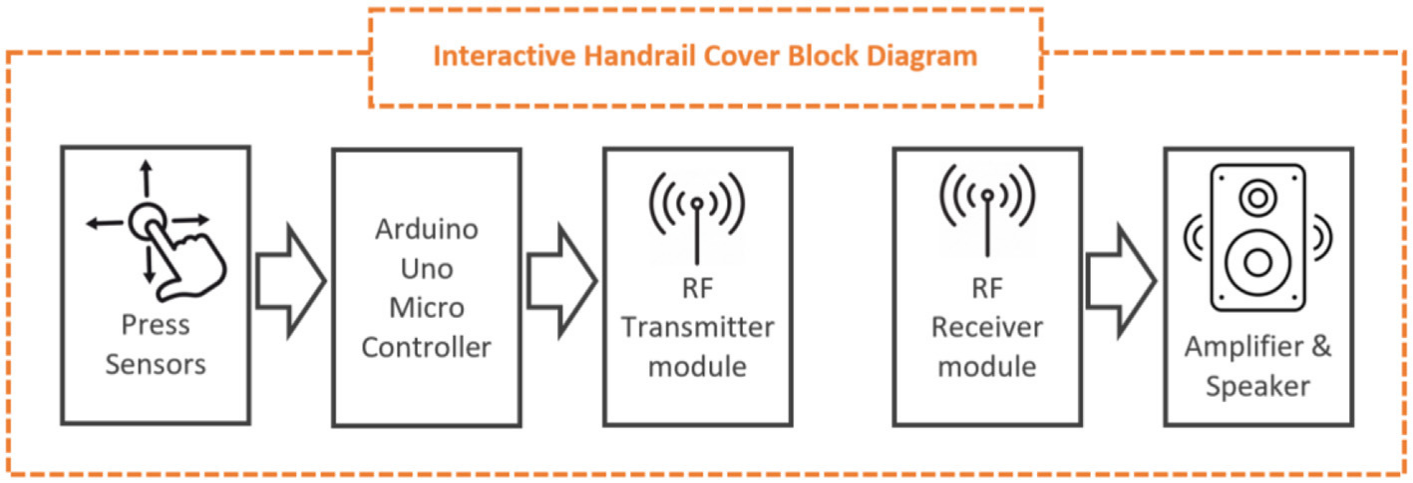
Interactive handrail cover block diagram.

**Figure 11:**
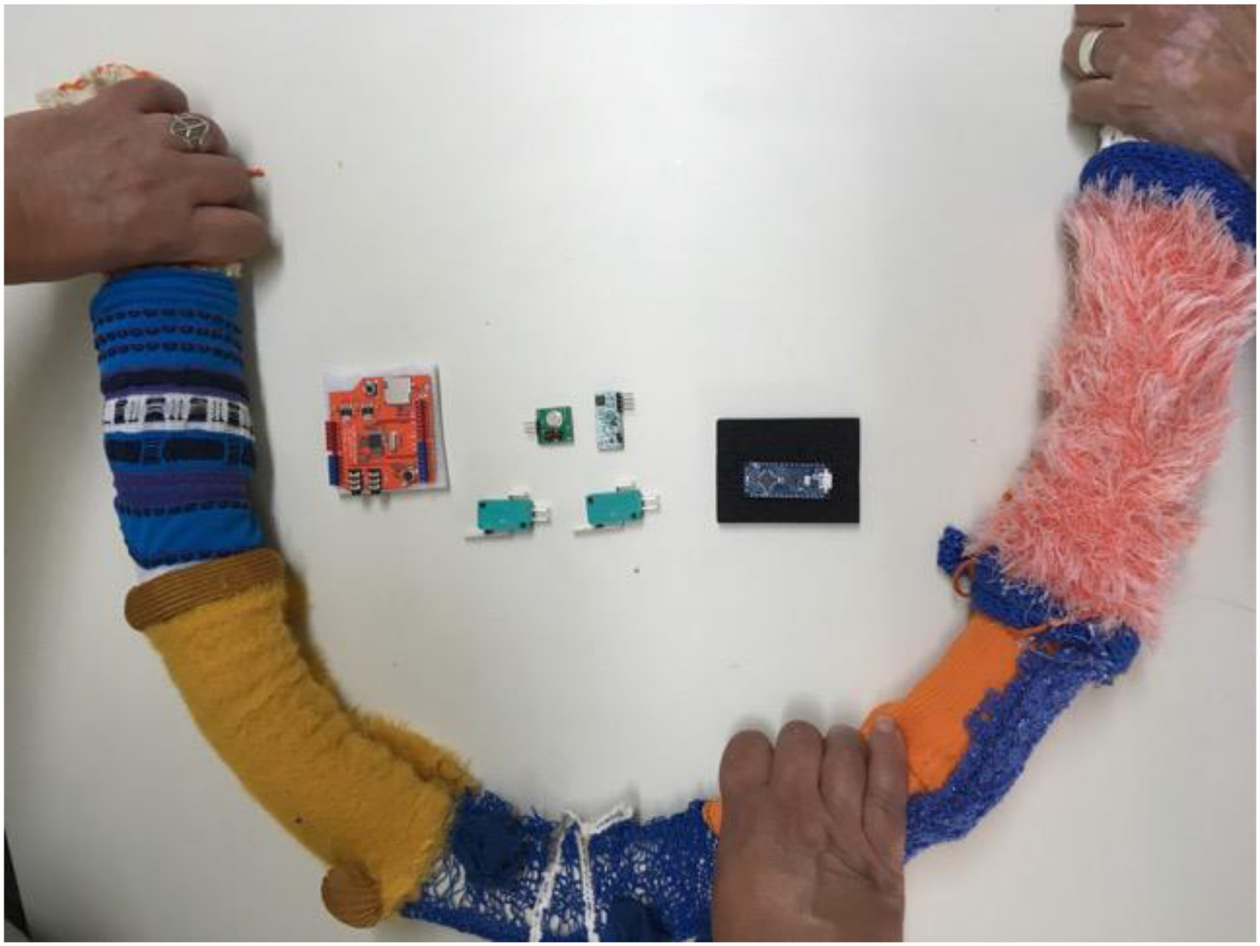
Interactive handrail prototype (with wireless speaker), work in progress.

An *interactive portable cover for a handrail* (Figures 10, 11). The interactive handrail cover is a cylindrical, flexible structure that can be placed on a handrail held by people as they walk in the hallway. The initial prototype incorporates three independent push buttons at various locations that detect a person's grip and wirelessly activate an external room speaker. When someone moves around the room while holding the handrail, they automatically push the buttons in sequence, producing a series of sounds. A pre-recorded story is embedded in an audio feature on the handrail, parts of which are played when the buttons are activated by the passing user. The concept for the function of this prototype is that on the full length of the handrail, with numerous push buttons, the narration of a whole story can take place. The health professionals in the LTC settings have suggested installing the cover on the handrail leading to a balcony, thus allowing the user to reach the outside space of the care home. There was also a suggestion that the chair could be located on this balcony to create meaningful links between the person's activity and the spatial objects.

The spatial prototypes were designed with a view to future production. For this reason, low-cost common commercial electronic components and the popular Arduino microcontrollers were used (65, 66). This practice has been successfully employed by the research team in a variety of previous research projects (67–69).

## 5 Discussion

Homing wellness explores the potential of the built environment to impact the quality and outcomes of dementia care. The built environments and the infrastructure technology within them affect the users, especially in healthcare environments, including living environments for people with dementia. The research aims to find appropriate ways to enhance the built environment and to provide evidence of its built environment as a communication enabler with therapeutic potential for people living with dementia.

To date, this research has investigated how playful spatial objects can be designed and produced in order to be incorporated into LTC settings for people with dementia, aiming to transform the space into a narrative agent. Three such prototypes have been designed and produced in partnership with health professionals in dedicated LTC settings. These prototypes have been presented to the partner care homes, and positive comments have been received on the prototypes' functionality and potential impact to benefit the users through their sensory attributes.

The research has accomplished the following:

a. The design and production of interactive spatial objects with narrative attributes that comply with the guidelines resulting from the action research Cycle 1/Phase 5.

The objects are accessible, ergonomic, and safe. The design choices deriving from the participatory process led to objects with familiar forms and features that could support feelings of homeliness with significant meaning and can be utilized in everyday routines. It was essential that the objects retain functionality as a primary feature to acquire meaning both as utility objects and storytelling tools. By using scent, sound, and light, but also tactile elements, they offer a multi-sensory experience, thus supporting the continuing use of the senses (70, 71). These sensory attributes and the use of actuators such as motors and fans provide triggers for interaction. The pleasant textures, familiar soothing sounds, and possibilities of favorite music choices can offer a sense of comfort and engagement opportunities (57, 72). Even though the objects incorporate advanced technology, their user interface aims to be intuitive, easily controllable, and provide a stress-free experience suitable for people in all stages of dementia (73, 74).

With no need for the presence of carers or activity coordinators when interacting, these objects could enhance a sense of empowerment and agency (35). Specific themes have been selected that can narrate imaginative or familiar stories. As an example, the armchair was designed to evoke the seaside experience; its textures, colors, and sounds can trigger relevant storytelling. This narrative, interactive nature of the objects could provide opportunities for communication and social contact between people with dementia and their carers and family members.

b. The incorporation of the spatial objects within the care home environment.

The participatory process led to design choices for the spatial objects that can be integrated into the care home environment. All objects are aesthetically modified versions of objects that could be found in such an environment, thus combining the ability to attract interaction both through their familiar features and their exhibiting playfulness.

c. The interrelation of the spatial objects, thus achieving a comprehensive narrative landscape.

Initial ideas for the combination of the spatial objects, when installed in the care homes, have already been examined through discussions with health professionals. The installation of the objects in the LTC settings would enable their interrelation, thus expanding the narrative ability of the space. Possibilities of meaningful links that could be created between the spatial objects depending on their location within the space and the walkways leading from one to the other will be explored together with people with dementia during the next stage of the project. For example, the tactile handrail cover, which can narrate parts of a story encouraging exploration and movement, could be placed on the corridor handrail that leads to one of the care homes' balconies. The specific balcony overlooks the sea, and the sea-themed armchair could be installed there to offer a multi-sensory sea experience to people who are not able to go outdoors. The spatial objects, incorporated in such an interrelated way in the care home-built environment, could stimulate engagement and communication through storytelling, thus transforming the space into a powerful narrative tool.

The research aims to benefit the wellbeing of people with dementia and contribute to the fight against the stigma and marginalization of people living with dementia. Homing Wellness can contribute to expanding knowledge in design for dementia by examining the narrative possibilities of the care home environment. It will also enhance the knowledge of dementia product design and create design guidelines and examples for best practices in dementia LTC settings.

## 6 Conclusion, implications, and future studies

It is possible to achieve the design and production of spatial objects for people with dementia following guidelines derived from existing literature and guidance from health professionals and people living with dementia. The combined use of embedded technology and textile-based materials, with attention to aesthetics, can lead to the production of exciting spatial objects that could engage people living with dementia. An interdisciplinary approach and collaboration between the researchers, the people with dementia, and health professionals can successfully drive the production of interrelated objects with storytelling features that may attribute narrative agency to dementia LTC settings. It is anticipated that spatial objects offering a sequence of visual, acoustic, olfactory, and tactile stimuli will activate a sensory environment that will be both soothing and exciting for people living with dementia. This environment might offer engagement, pleasure, and fun, so people with dementia can have a good time, communicate in an embodied way, and feel creative, validated, and celebrated in their living environment.

The spatial objects will be installed in the partner LTC settings so that the next stage of the research can be carried out. During this stage, people with dementia will evaluate the prototypes. There may be additional action research cycles in future, depending on the results and conclusions of the evaluation, aiming to further develop the prototypes with co-design workshops with people living with dementia. The findings will be discussed in relation to findings from previous studies and will be compared. Suggestions for further research will be discussed.

## Data availability statement

The original contributions presented in the study are included in the article/supplementary material. Further inquiries can be directed to the corresponding author.

## Ethics statement

The studies involving human participants were reviewed and approved by the Research Ethics Committee of the University of West Attica, Athens, Greece (approval ref: AP.ΠP*Ω*T: 32538 - 24/03/2023). Written consent of the partner care homes was obtained to participate in the research. Their participation involves consultation with their health professionals and also the participation of people living with dementia. People living with dementia will be provided with individual consent forms. The stage specified in this manuscript outlines consultations with the professionals, however the participation from the residents will come at the second stage of research.

## Author contributions

LM was responsible for the organization of the project, the field testing, and the writing of the manuscript. AC was responsible for supervising and implementing the technical part of spatial object development and the writing of the manuscript. AT was responsible for implementing the technical part of the spatial objects' development and writing the manuscript. GP was the principal investigator of the project. JvH provided scientific consulting on the project and reviewed the manuscript. ES was responsible for developing and implementing the integrated textiles and reviewing the manuscript. ZG and ST helped with the development of the product design and the environmental arrangements and reviewed the manuscript. CD contributed to the technical part of the spatial objects' development and reviewed the manuscript. All authors contributed to the article and approved the submitted version.
